# Vertical Organic Electrochemical Transistors and Electronics for Low Amplitude Micro‐Organ Signals

**DOI:** 10.1002/advs.202105211

**Published:** 2022-01-22

**Authors:** Myriam Abarkan, Antoine Pirog, Donnie Mafilaza, Gaurav Pathak, Gilles N'Kaoua, Emilie Puginier, Rodney O'Connor, Matthieu Raoux, Mary J. Donahue, Sylvie Renaud, Jochen Lang

**Affiliations:** ^1^ UMR CNRS 5248 (CBMN, Chemistry and Biology of Membranes) Univ. Bordeaux Av Geoffroy St Hilaire Pessac F‐33600 France; ^2^ UMR CNRS 5218 (IMS, Integration of Materials into Systems) Univ. Bordeaux Bordeaux Institut National Polytechnique 351 Cours de la Libération Talence F‐33405 France; ^3^ Department of Bioelectronics Mines Saint Etienne CMP‐EMSE MOC Gardanne 13541 France; ^4^ Linköping University Department of Science and Technology (ITN) Laboratory of Organic Electronics Linköping SE‐581 83 Sweden

**Keywords:** biosensor, cardiomyocytes, diabetes, electrophysiology, insulin, organic electrochemical transistors, pancreatic islets

## Abstract

Electrical signals are fundamental to key biological events such as brain activity, heartbeat, or vital hormone secretion. Their capture and analysis provide insight into cell or organ physiology and a number of bioelectronic medical devices aim to improve signal acquisition. Organic electrochemical transistors (OECT) have proven their capacity to capture neuronal and cardiac signals with high fidelity and amplification. Vertical PEDOT:PSS‐based OECTs (vOECTs) further enhance signal amplification and device density but have not been characterized in biological applications. An electronic board with individually tuneable transistor biases overcomes fabrication induced heterogeneity in device metrics and allows quantitative biological experiments. Careful exploration of vOECT electric parameters defines voltage biases compatible with reliable transistor function in biological experiments and provides useful maximal transconductance values without influencing cellular signal generation or propagation. This permits successful application in monitoring micro‐organs of prime importance in diabetes, the endocrine pancreatic islets, which are known for their far smaller signal amplitudes as compared to neurons or heart cells. Moreover, vOECTs capture their single‐cell action potentials and multicellular slow potentials reflecting micro‐organ organizations as well as their modulation by the physiological stimulator glucose. This opens the possibility to use OECTs in new biomedical fields well beyond their classical applications.

## Graphical abstract

Islet micro‐organs are of prime importance in health and diabetes but their electrical signals are far smaller than those of brain or heart. High signal amplification by vertical organic electrochemical transistors (vOECTs) combined with a tunable electronic board allows reliable quantitative capture of glucose‐dependent single‐cell and multicellular islet activities. This opens new biomedical applications for OECTs beyond their classical use.

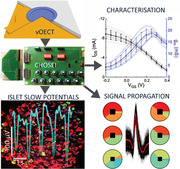

## 1 Introduction

Electrical signals in cells and micro‐organs provide the base for key biological events such as brain activity, heartbeat, or vital hormone secretion. Their capture allows not only crucial insight into physiological phenomena but also opens the possibility to develop diverse biosensors for continuous monitoring and consecutive therapy.^[^ ^1^, ^2^ ^]^ Electrical signals are generated by single cells as action potentials and also by cell groups, regions, or micro‐organs as field potentials in defined regions or micro‐organs that can be recorded extracellularly.^[^ ^3^ ^]^ Concomitant multi‐parametric analysis of these electrical signals not only provides insight into the activity of a given cell but also informs about higher organizational modes.^[^ ^4^, ^5^ ^]^ Although extracellular recording configurations do not provide the same richness in information as intracellular recordings, this approach keeps the biological substrate intact, does not disturb metabolic events underlying or shaping electrical activity and permits long‐term recordings necessary to understand physiological function and for the development of biosensors.

Electrical signals offer several advantages as compared to other activity read‐outs. Indeed, electrical signals are easier to analyze and quantify and moreover, in contrast to imaging, far higher sampling rates are feasible and optical probes are not required. This avoids problems such as the use of transgenics or organic molecules with inherent difficulties in their tissue or micro‐organ penetrance and potential genetic bias as well as ensuring general applicability in human tissue. Fluorescence bleaching or heat generation is not an issue in recording electrical signals and all components are well suited for miniaturization.^[^ ^6^ ^]^ The signal to noise ratio (SNR), however, poses a major issue in electrical recordings. Although this may be less prominent in neurons or cardiomyocytes which are endowed with depolarizations of considerable amplitude, other vital cells of the body, such as endocrine cells, depolarize only to far smaller amplitudes.^[^ ^7^ ^]^ Coating metal electrodes with the conducting polymer poly(3,4‐ethylenedioxythiophene) polystyrene sulfonate (PEDOT:PSS) offers some improvement in extracellular recordings, however the recording of biologically important small action potentials by MEAs remains difficult.^[^ ^5^, ^8^ ^]^ Some transistor technologies offer an attractive means to address this problem as signals are amplified directly at the source by their intrinsic voltage‐to‐current conversion, thus reducing noise in contrast to classical metal electrodes.^[^ ^9^ ^]^ The recent developments in organic electrochemical transistors (OECTs) offer unprecedented versatility in terms of fabrication methods, sensor geometry, miniaturization, stability in aqueous environments, cell or tissue interaction, and low‐cost printing.^[^ ^10^ ^]^ The geometry of the OECT itself also profoundly influences the behavior of the transistor and notably vertical OECTs (vOECT) exhibit very high transconductance as well as good cut‐off frequencies.^[^ ^11^, ^12^ ^]^ Moreover, the vOECT geometry favors device density, an important advantage in future miniaturization and development of high‐density arrays for improved spatial resolution.

OECTs are promising tools for fundamental research and as components of biosensors or biomedical devices. Their remarkable characteristics have been used in the field of classical bioelectronics, that is, brain or heart recordings, to gain insight using EEG‐ or ECG‐like configurations, and taking advantage of their favorable biocompatibility and form factor.^[^ ^13^ ^]^ Moreover, successful uses of OECTs have been reported for neural probes as well as in cellular recordings of cardiomyocytes, yet some important issues remain to be addressed.^[^ ^14^, ^15^, ^16^, ^17^, ^18^, ^19^, ^20^, ^21^, ^22^ ^]^ Long‐term quantitative observations of living material rely on the assumption of operational stability of OECTs over a prolonged period of time. While physical stability has been reported previously this issue has often not been addressed quantitatively for prolonged polarized states with few exceptions.^[^ ^16^, ^23^, ^24^, ^25^ ^]^ Moreover, the fabrication of OECT multichannel devices entails some variation between the probes, which have to be controlled or equalized. For example, this may be achieved via corresponding calibration of the electrical circuits to provide meaningful quantitative read‐outs in long‐term experiments. To account for transistor properties, a parameter extraction methodology is required. Finally, electrogenic cells generate signals of different amplitudes. Neurons or cardiomyocytes depolarize to considerably larger values (+40 mV) than endocrine cells, such as the islets (0 mV), required for nutrient homeostasis and a major player in diabetes.^[^ ^7^, ^26^, ^27^ ^]^ Here we demonstrate the possibility to fully exploit the potential of OECTs in fundamental research and potential biomedical applications through their use with micro‐organs such as the islets, which are inherently far more difficult to monitor.

## 2 Results

### 2.1 Chip Geometry and Vertical OECT Electrical Performance

**Figure 1:**
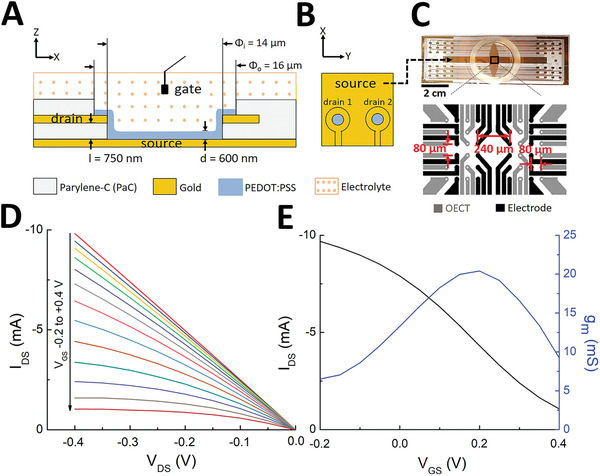
Structure and physical performance of vertical PEDOT:PSS OECT devices. A,B) Cross‐sectional view (A) and top view (B) demonstrating the layout of the transistor with a common source but individual drains and the layers dimensions: l, the parylene‐C layer thickness between drain and source contacts (750 nm); d, the PEDOT:PSS layer thickness (600 nm). *Φ*i and *Φ*o, inner well and outer well dimensions, respectively. An Ag/AgCl gate electrode inside the electrolyte solution is used for each experiment. C) Photograph of the device (scale bar: 2 cm) with the common source visible (see arrow) and layout showing 12 OECTs and 12 electrodes in each side of an OECT array as well as dimensions. D) Output characteristics of a vertical PEDOT:PSS transistor in physiological solution showing the drain current I_DS_ as a function of drain voltage V_DS_ ( = −0.4 V) for gate voltages VGS varying from −0.2 to 0.4 by 0.05 V steps. E) Transfer curve and resulting transconductance at V_DS_ = −0.4 V.

The vOECTs used here have source and drain gold contacts in different planes (**Figure** **1**). This vertical configuration allows arranging a higher number of transistors in a given geometrical area, thus increasing the spatial resolution (Figure 1A,B). The maximal transconductance, *g*_max_, which defines amplification potency, is inversely related to channel length, which can be considerably reduced to sub‐micrometer dimensions in the vertical arrangement.^[^ ^11^, ^12^ ^]^ As shown in Figure 1B, our chip consisted of 12 vOECT channels and 12 electrodes on each side of the midline of the device (for details on electrodes, see Figure S1, Supporting Information). Steady‐state characterization of the transistors was performed by measuring vOECT output and transfer characteristics (Figure 1C,D), demonstrating p‐type characteristics with the expected excellent maximal transconductance *g*_max_ of ≈20 mS as reported previously.^[^ ^12^ ^]^

### 2.2 Electronic Board for Data Acquisition

**Figure 2:**
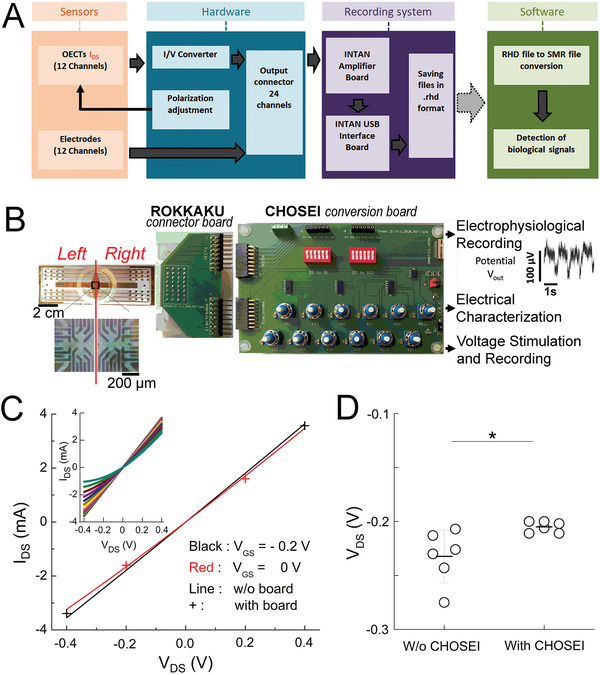
Electronic board for data acquisition. A) Block diagram of the acquisition process and data flow. The designed hardware connects the sensor device, allows voltage bias application to the transistors, adjusts the drain–source voltage bias, and performs the conversion of drain currents from the OECTs into voltage signals. Recording files, saved in INTAN format (.rhd) are converted to Spike2 format (.smr) for analysis. B) Working setup for recording: system hardware components and their connections to record 24 OECT channels simultaneously. The device‐specific connector board ROKKAKU connects all OECTs and electrodes to the CHOSEI board which permits coarse and fine tuning of the drain–source voltage bias for each channel and converts *I*_DS_ to an analyzable voltage signal. C) Output characteristics of the board with the transistor drain current, *I*_DS_ as a function of negative and positive drain voltage, *V*_DS_ ( = −0.4 and −0.4 V) for a gate voltage, *V*_GS_ ( = −0.2 and 0 V). Inset: output characteristics of the transistor with the drain current, *I*_DS_ as a function of negative and positive drain voltage, *V*_DS_, for a gate voltage, *V*_GS_ from −0.2 to 0.4 V. *V*_DS_ corresponds to the value indicated by the Agilent voltmeter (Figure S2E, Supporting Information) and was obtained by common coarse tuning (via the J8 jumper), then individualized fine tuning via the corresponding potentiometers connected individually to each of the load resistors until the desired *V*_DS_ was obtained. D) Drain–source voltage bias (here set to *V*_DS_ = −0.2 V) of OECT channels with or without adjustment by CHOSEI. Means ± SEM; Mann–Whitney test; *2*p* < 0.05; *N* = 6.

Multichannel hardware is currently not commercially available to connect sensor devices, provide transistor voltage bias, and convert OECT drain currents into readable voltage signals. We therefore developed a custom board which also addresses variability among channel transconductances that may interfere with interpretation of analyzed biological signals. For this reason we included individually tunable drain–source voltage biases to gain homogeneity (**Figure** **2**A). Adding a device specific connection board, which we termed ROKKAKU, allows adaptation to different OECT chip layouts, and fabrications schemes. The connection board matches the positions of all OECTs and electrodes present on a sensor device to record all signals simultaneously (Figure 2B).

Subsequently, a polarization and conversion board, named CHOSEI, allows the conversion of currents measured by the OECTs to voltages for further acquisition by conventional acquisition hardware (here INTAN) through means of a 560 Ω load resistor, as well as the adjustment of the drain–source polarization voltage for each OECT channel (Figure 2B, Figure S2, and Table S1, Supporting Information). An output connector with 24 pins on the board plugs connects the OECTs and electrodes to an INTAN recording system. Importantly, ROKKAKU/CHOSEI can be used for vOECT characterizations, stimulations, and electrophysiological recordings. Details of the setup and use in various experiments are given in Figure S2 and Table S1, Supporting Information.

The output characteristics of the transistor were determined as shown in Figure 2C. The boards did not distort by saturation or non‐linearity the observed drain current *I*_DS_ as a function of voltage *V*_DS_ for the tested gate voltages *V*_GS_. To address channel‐to‐channel variations in performance and to permit a uniform *V*_DS_ for all OECT channels on a given array, we adjusted the drain–source voltage bias of each OECT channel to the same *V*_DS_ via the CHOSEI potentiometers.

As shown in Figure 2D the application of a supply voltage common to all load resistors results in a considerable scattering of *V*_DS_ by ≈30% between the extrema. In contrast, individualization of supply voltages by fine tuning with CHOSEI's potentiometers biased every OECT at *V*_DS_ values that varied only by 5% (Figure 2D). A variation of 30% can substantially alter quantitative read‐outs in terms of recorded cellular signals (for example see variation of *V*_DS_ −0.1 versus −0.2 V in Figure 5B). We also compared the noise of resistors of values equivalent to the drain–source junction of the vOECTs in the dry vOECTs or in the wet setup of vOECTs or electrodes seeded with HL‐1 cells (Figure S3, Supporting Information). Thus, the board will provide truly comparable read‐outs in terms of amplitude without adding additional noise and maximum sensitivity is not limited by transistor noise. Subsequently we tested our electronic setup by using simulated biological signals by imposing electric pulses via an electrode present on the chip and recording either via electrodes or via vOECT channels (Figure S4, Supporting Information). The output signals recorded with OECTs clearly have signal‐to‐noise ratios superior to those captured by electrodes.

### 2.3 Electrical Performances and Stability of the Vertical OECTs

In order to evaluate whether vOECTs can be used for cell or micro‐organ recording and we measured the electrical performances in KCl solution, physiological buffered salt solution as well as culture medium containing serum, without or with coating of devices with extracellular matrix that improves cell adhesion. The vOECTs were stable for up to 10 days during these short‐term measurements at *V*_GS_ from −0.2 to 0.4 V (Figure S5, Supporting Information).

**Figure 3:**
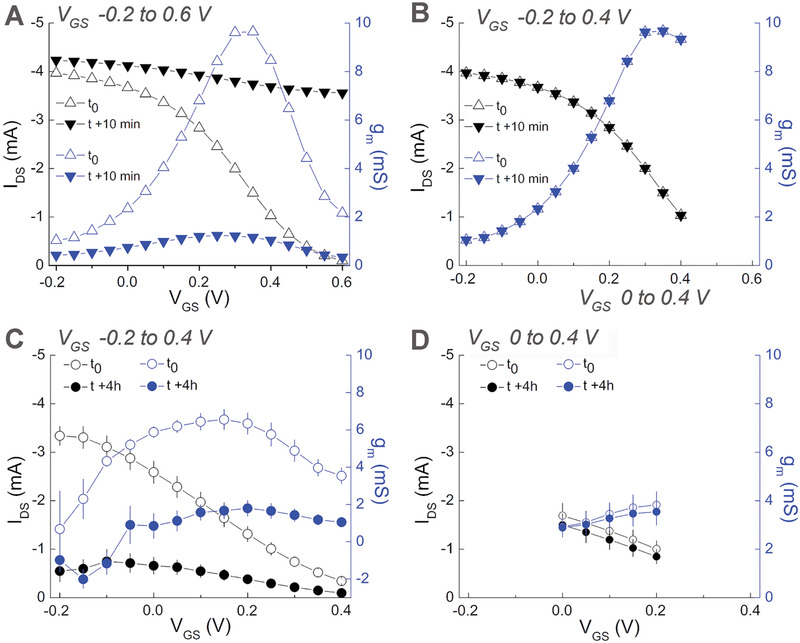
Influence of voltage bias on the stability of vertical OECTs performances in physiological buffered salt solution. A) Transfer curves and resulting transconductances for two consecutive measurements at *V*_DS_ −0.4 V, for *V*_GS_ varying from −0.2 to 0.6 at 0.05 V steps. Measurements were separated by 10 min pause under constant *V*_DS_ −0.4 V. B) Analogous conditions same as (A) but *V*_GS_ varying from −0.2 to 0.4 V. C) Analogous conditions as in (A) but varying *V*_GS_ from −0.2 to 0.4 V and measurements after 4 h of constant polarization. D) Same as in (A) but varying *V*_GS_ only from 0 to 0.2 V at *t*_0_ (initial measurement) or after 4 h of constant polarization. *N* = 6, means + SEM. Experimental details see also Figure S5, Supporting Information.

Meaningful biological experiments require recordings over hours at least, so we consequently evaluated the stability of the vOECTs for consecutive measurements comparing ranges of bias voltages and maintain drain–source polarization in between measurements (**Figure** **3** and Figure S5, Supporting Information). The transfer curve and the transconductance at *V*_DS_ −0.4 V, for *V*_GS_ varying from −0.2 to 0.6 V are considerably decreased during a second measurement after 10 min constant bias at *V*_DS_ = 0.4 V (Figure 3A), as compared to a narrower range of *V*_GS_ varying only from −0.2 to 0.4 V (Figure 3B). However, using a longer active time range of 4 h, electrical performances for *V*_GS_ varying from −0.2 to 0.4 V were also decreased and full stability was attained only when *V*_GS_ variation were reduced to 0 to 0.2 V (Figure 3C,D).

**Figure 4:**
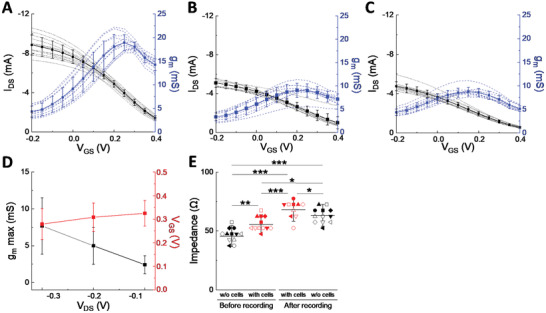
Performance and Stability of vertical OECTs before, during, and after culture of HL‐1 cardiomyocytes. A) Transfer curve and resulting transconductance at *V*_DS_ 0.4 V, for *V*_GS_ varying from −0.2 to 0.4 V in physiological buffered salt solution before seeding cells on the vOECTs array, *N* = 12. B) In culture medium with HL‐1 cardiomyocytes seeded on vOECTs array, *N* = 10. C) In physiological buffered salt solution after removal of HL‐1 cardiomyocytes, *N* = 10. D) Summary plot of different imposed drain voltages on *g_m_* max and *V*_GS_ with HL‐1 cells in culture medium on OECTs array, *N* = 10. E) Impedance of vOECTs without cells (in physiological buffered salt solution) and with cells (in culture medium) before electrophysiological recordings and afterwards first with cells attached and then after removal of cells. *N* = 10–12. Given are means ± SEM; ANOVA and Tukey's post‐hoc analysis; *2*p* < 0.05, **2*p* < 0.01, ***2*p* < 0.001.

Subsequently we used this gate range to evaluate vOECTs with cells for stability. For these experiments we employed first the established cardiomyocyte‐like cell line HL‐1 as a model since OECT recordings with such cell types have been published previously.^[^ ^16^, ^17^, ^18^, ^28^, ^29^ ^]^ Moreover, these atrial cardiac muscle‐derived HL‐1 cells are well known to maintain the cardiac‐specific phenotype and action potentials during the culture period.^[^ ^30^ ^]^ The vOECTs used in this series of experiments were characterized before cell seeding, during culture of HL‐1 cells on the chip, and after removal of cells. To evaluate the uniformity of maximal transconductance *g_m_* all channels of an array were measured (**Figure** **4**). The *g_m_* for all vOECT channels was stable and amounted to ≈20 mS before cell culture, that is, in the presence of physiological buffered salt solution. Culturing the HL‐1 cells for 6 days led to a uniform reduction of *g_m_* to ≈10 mS. We attribute these changes to the adherent confluent layer of cardiomyocytes, which introduce an additional resistance in the electrical circuit and impede the diffusion of ions.^[^ ^31^, ^32^, ^33^, ^34^ ^]^ The observed reduction persisted after the removal of cells, probably due to the known presence of residual proteins (Figure 4A–D).

As *g_m_* does not change between culturing cells and after removal, vOECTs can be reused if specific electrical conditions are applied, as permitted by the electric board described above. The impedance of each OECT channel before recording (with and without cells) and after recording experiments (Figure 4E) was in line with differences introduced by characterization in the absence of cells, some decrease in performance in the presence of cell layers, and some deterioration due to usage. Note that cell density does not change during the short experiment time.

### 2.4 Monitoring Electrical Activity of HL‐1 Cardiomyocytes

**Figure 5:**
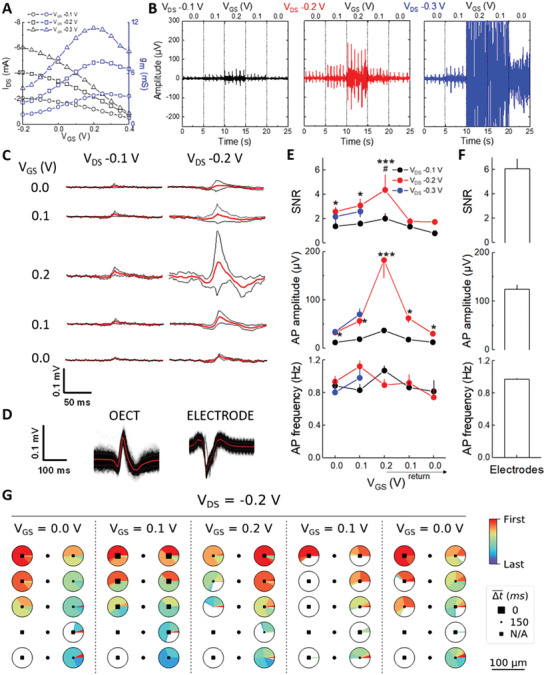
Recording of HL‐1 cardiomyocytes with vOECTs. A) Transfer curves and resulting transconductances of OECTs array covered with HL‐1 cells at *V*_DS_ and *V*_GS_ values used for recording of action potentials. B) Recorded spontaneous action potential at different *V*_DS_ and a sweep of *V*_GS_. 5 s of 15 min recordings per condition are shown. At *V*_DS_ −0.3 V, *V*_GS_ 0.2 V damage to HL‐1 cell layers was observed. C) Shapes of action potentials observed at conditions given in the left and middle panel of (B). Means, red lines; standard deviations, black; *n* = 8 channels. D) Comparison of extracted mean configuration of HL‐1 action potentials observed by vOECTs (*V*_DS_ −0.2 V, *V*_GS_ 0.2 V; *n* = 253 AP) versus electrodes (MEA; *n* = 314 AP); red, means; black, standard deviations. For filters used, see Figure S7, Supporting Information. E) Top panel: SNRs of conditions used in (B) to (D). Middle panel: Action potential amplitudes of HL‐1 cells evolved at different *V*_DS_/*V*_GS_ with a maximum at *V*_DS_ −0.2 V, *V*_GS_ 0.2 V. Lower panel: Action potential frequency of HL‐1 cells at different *V*_DS_/*V*_GS_ remained stable. Means ± SEM; ANOVA, Tukey's analysis; *V*_DS_ −0.2 V versus *V*_DS_ −0.1 V, * 2*p* < 0.05, *** 2*p* < 0.001; *V*_GS_ 0.2 V versus other *V*_GS_ at same *V*_DS_, ^#^2*p* < 0.05; *N* = 8. F) Analysis of recordings via electrodes on the same devices, *N* = 3. G) Analysis of action potential propagation across the vOECT chip at *V*_DS_ −0.2 and indicated *V*_GS_ sweep. The pie charts indicate the relative occurrence of being first or last action potential in a series of events (color code at the right). The size of solid squares in the pie chart indicates the mean time delay of the action potentials.

To investigate the stability of the biological preparation on the array along with various voltage biases, cardiac cells were used first. The action potentials were measured after 6 days of culture on vOECTs to ensure spontaneous electrical activity. Transfer curves and the resulting transconductance values of vOECTs covered with HL‐1 cells indicated *g_m,_max* at *V*_GS_ = 0.2 V, which increased from *V*_DS_ −0.1 to −0.3 V (**Figure** **5**A). We gradually tested these different ranges of polarization with increasing/decreasing sweeps of *V*_GS_ from 0 to 0.2 V and representative traces are given in Figure 5B. At *V*_DS_ = −0.3 V and, *V*_GS_ = 0.2 V signals from HL‐1 cells were lost and the confluent cell layer was disrupted. We believe that this occurred subsequent to damage of the vOECT above *V*_DS_ = −0.3 V/*V*_GS_ = 0.1 V as upon return to *V*_DS_ −0.3 V/*V*_GS_ 0.0 V only large noise was recorded which is not consistent with loss of vOECT‐cell contact only. To reliably extract action potentials, filters were chosen by parametric analysis. The detection of action potentials by vOECTs or electrodes was robust over a large range of the adaptive threshold *σ* with a high‐pass filter of 10 Hz and low pass filter of 100 Hz (Figure S7A,B, Supporting Information). As cardiomyocyte action potentials are regularly spaced in time, we could also evaluate their frequency from the interspike interval (ISI) by identifying a Gaussian distribution (Figure S7C,D, Supporting Information). The shape and amplitude of action potentials under the different electric conditions is given in Figure 5C and although obviously their amplitude differed, kinetics remained comparable. The mean shape of action potentials at *V*_DS_ −0.2 V and *V*_GS_ 0.2 V (Figure 5D) represents the precise inverse of action potentials captured by electrodes on the same chip.

As cardiomyocyte action potentials are regularly spaced in time, we could also evaluate their frequency from the interspike interval (ISI) by identifying a Gaussian distribution (Figure S7C,D, Supporting Information). The shape and amplitude of action potential under the different electric conditions is given in Figure 5C and although obviously their amplitude differed, kinetics remained comparable. The mean shape of action potentials at *V*_DS_ −0.2 V and *V*_GS_ 0.2 V (Figure 5D) represents the precise inverse of action potentials captured by electrodes on the same chip.

Electrophysiological signals are measured by OECTs as current fluctuations (*I*_DS_) which are in turn converted to a potential using our developed electronic boards, whereas electrodes directly sense the potential. In this series of experiments, the SNR of HL‐1 APs is between 3 and 6 at optimal conditions (Figure 5E). Importantly, the frequency of action potentials (≈1 Hz) did not change during the different electrical conditions (Figure 5E), whereas the amplitude was clearly most prominent at *V*_DS_ −0.2 V and *V*_GS_ 0.2 V and changed according to maximal transconductance (Figure 5B,E). Note that values for *V*_GS_ −0.3 V are only given up to *V*_DS_ 0.1 V due to transistor breakdown (see Figure 5B). Comparison to electrodes on the same devices indicated similar SNR for PEDOT‐covered electrodes, whereas mean amplitudes were clearly lower (Figure 5F). The signal shape of action potentials is determined by the different expression levels of several ion channels in the cell membrane and the apparent signal amplitude is mainly influenced through the cell coverage by the sensor and the cell/sensor resistance. Stability in action potential shape and frequency strongly indicate that the electrical parameters used here do not influence the biological behavior of the cells. Finally, we evaluated the propagation of action potentials across the vOECT channels on the chip. The maps show a stable direction of action potential propagation at *V*_DS_ −0.2 (Figure 5G) or −0.1 V and *V*_DS_ −0.3 V (Figure S5E, Supporting Information) before cell and/or vOECT damage occurred in the latter condition. We also observed a well‐known rhythmicity of action potentials as well as their sensitivity to nor‐epinephrine and the calcium channel blocker nifedipine (Figure S8, Supporting Information).

### 2.5 Monitoring the Activity of Endocrine Pancreatic Islets

**Figure 6:**
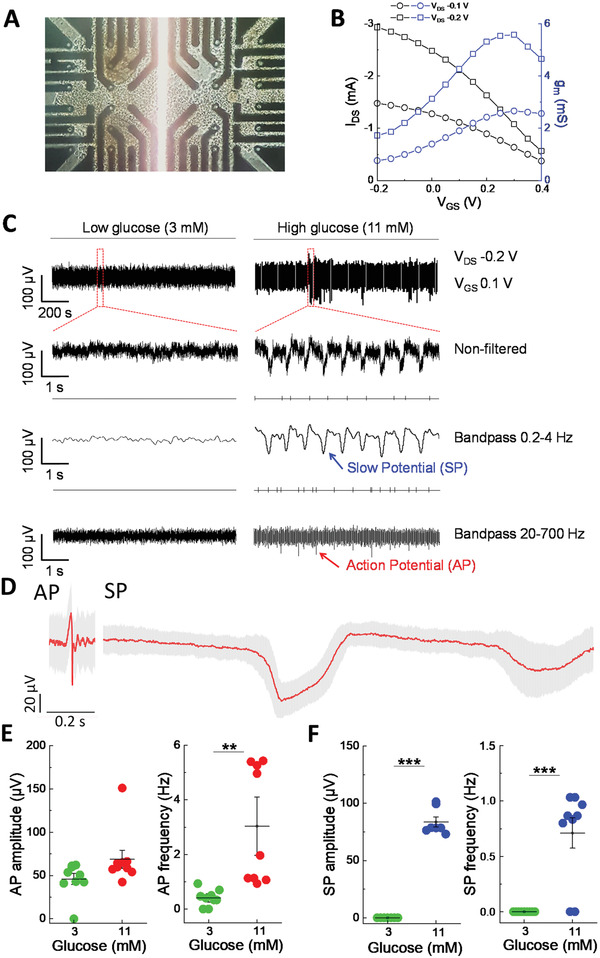
Recording of endocrine islet cells on vOECTs. A) vOECT Array with pancreatic islet cell clusters. B) Transfer and transconductance curves at *V*_DS_ −0.1 and −0.2 V at *V*_GS_ −0.2 to 0.4 V of vOECTs with islets in culture medium. C) Representative raw and filtered recordings of islets at low glucose (non‐stimulatory, 3 mm) and high glucose (stimulatory, 11 mm) in physiological buffered ion solution at *V*_DS_ −0.2 V and *V*_GS_ 0.1 V. The different time scales are shown as well as non‐filtered and band pass filtered traces (0.2–4 Hz, 20–700 Hz). The presence of slow potentials reflecting islet *β*‐cell coupling are indicated. D) Average AP and SP at 15 mm glucose (*V*_DS_ −0.2 V, *V*_GS_ 0.1 V; mean in red and SEM in gray; AP mean amplitude 69 µV, *n* = 303; SP mean amplitude 110 µV, *n* = 141). E,F) Action potential and slow potential amplitudes and frequencies at low glucose and high glucose stimulation (*V*_DS_ −0.2 V, *V*_GS_ 0.1 V). Means ± SEM; paired *t*‐test; **2*p* < 0.01, ***2*p* < 0.001; *N* = 9.

After validating our electronic board and establishing the stable drain–source and gate–source voltage bias region, we addressed the recording of a more difficult biological sample on vOECTs, that is, pancreatic islets. These primary micro‐organs are non‐proliferating and known for action potentials of far smaller amplitude than cardiomyocytes and as a primary micro‐organ they are more demanding in terms of culture in contrast to cell lines. The characteristics of vOECTs before, during, and after culture were comparable to what was observed for clonal HL‐1 cells (**Figure** **6**A,B).

To test physiological function, islets on vOECTs were exposed to either 3 mm glucose, at which the main type of islet cells, that is, *β*‐cells, are known to remain rather inactive, or to 11 mm glucose where the intracellular metabolism of the sugar leads to *β*‐cell depolarization and secretion of the hypoglycemic hormone insulin.^[^ ^5^, ^35^ ^]^ Interestingly, dispersed islet cell clusters of these micro‐organs exhibit two types of electrical signals similar to neurons, action potentials generated by any single islet cells and so‐called slow‐potentials, a spatial summation of coordinated *β*‐cell activity and coupling.^[^ ^36^, ^37^ ^]^ Using a bias of *V*_DS_ −0.1 V/*V*_GS_ 0.2 V detected APs were well detected, whereas SPs were apparent upon inspection of traces but could not reliably be extracted (Figure S10A, Supporting Information). Action potentials were absent at low glucose (3 mm), but appeared at high glucose stimulation (11 mm) and similar to HL‐1 cell recordings, only action potential amplitude but not action potential frequency was altered by applying different biases (Figure S10B, Supporting Information). This suggests again that a change in biases alters the transconductance but does not alter the behavior of the cells. Observed frequencies were in line with previously published frequencies recorded with micro‐electrode arrays.^[^ ^5^, ^8^ ^]^ Using a bias of *V*_DS_ −0.2 V/*V*_GS_ 0.1 V, we observed both APs and SPs (Figure 6C) and high pass filter of 0.2 Hz and low pass filter of 4 Hz (4th order) can be used under this voltage bias to detect and extract robustly these SPs (Figure S11, Supporting Information).

We have also determined the mean shape of APs and SPs. APs were of ≈100 ms duration, similar as described for MEAs, and SPs were as expected of much longer duration. However, the mean AP amplitude on vOECTs was 69 µV, whereas only 12 µV has been published for islet recordings via MEAs.^[^ ^5^ ^]^ Both, APs and SPs, were glucose‐dependent and faithfully reflect nutrient‐induced islet activation (Figure 6E,F). Note that only AP frequency but not amplitude increased at stimulatory glucose levels, as can be expected from a unitary signal. In contrast, in the case of SPs both, frequency and amplitude, increased as the latter reflects electrical coupling between single *β*‐cells, a hallmark in the change of micro‐organ organization at stimulatory glucose levels.^[^ ^5^, ^36^ ^]^

**Figure 7:**
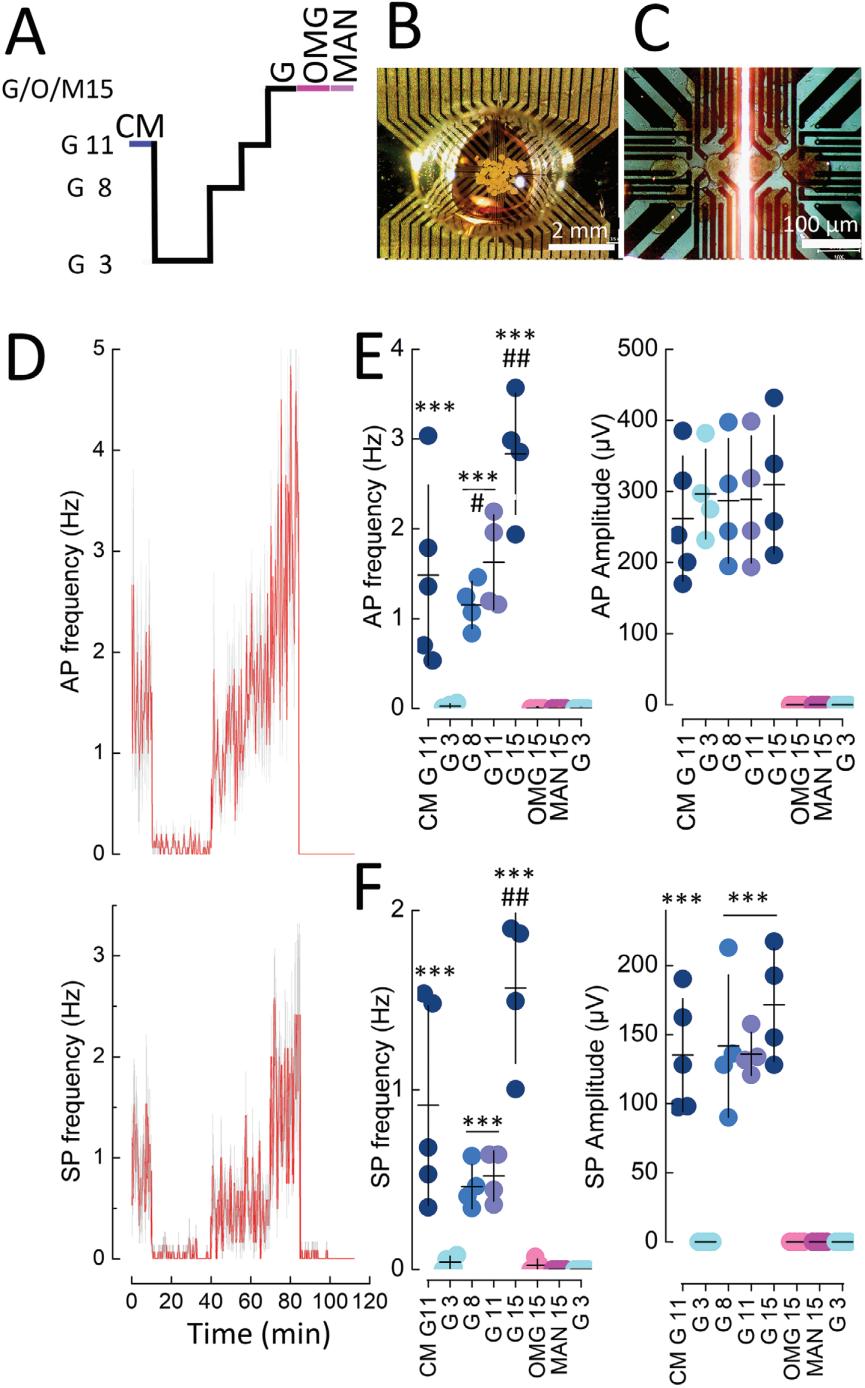
Recording of islet micro‐organs on vOECTs at different glucose concentrations and in the presence of non‐metabolizable sugars. A) Stimulation protocol with 3, 8, 11, or 15 mm glucose (G), in the presence of culture medium (CM; containing 11 mm glucose and amino acids) or in the presence of non‐metabolizable 15 mm 3‐ortho‐methylglucose (OMG) or 15 mm mannitol (MAN). B) View of islets seeded on vOECTs in a PDMS microfluidic well. C) Enlarged view of islets on vOECTs just prior to recording. D) Time course of action potential (AP) and slow potential (SP) frequencies during the stimulation protocol given in (A). Means in red, SEM in grey. E) Mean action potential frequencies and amplitudes during the indicated conditions. F) Mean slow potential frequencies and amplitudes during the indicated conditions. In (E) and (F) means (horizontal bar) and SEM (vertical bars) are indicated. Tukey post‐hoc test; ***2*p* < 0.001 versus G3, ^##^ 2*p* < 0.01 versus G11 or G8. ^#^, 2*p* < 0.05 versus G8. Glucose concentrations (G3–G15) and electrical activity were highly correlated (frequencies AP, *r* ^2^ = 0.9852, *p* = 0.004; frequencies SP, *r* ^2^ 0.8528, *p* = 0.03). Insulin secretion raised from 0.91 ± 0.19 ng/mL/15 min at G3 to 11.04 ± 0.18 ng/mL/15 min at G8 (2*p* < 0.001).

In order to investigate intact islet micro‐organs as well, we set up a simple microfluidic device on the OECTs to reduce liquid volumes and ensure sufficient channel coverage (**Figure** **7**B,C). A change from culture medium, containing 11 mm glucose, to electrophysiological buffer with 3 mm glucose, lead to a rapid drop in activity (Figure 7A,D). Subsequent exposure to 8, 11, and 15 mm glucose increased AP and SP frequencies in a dose dependent manner, whereas their amplitude remained stable as expected for unitary signals (Figure 7D–F). The increase in electrical activity was mirrored by increased insulin secretion. In contrast, the non‐metabolizable sugar 3‐O‐methyl‐glucose or mannitol did not elicit any electrical response excluding potential osmotic effects and underscoring the specificity of the recordings.

## 3 Discussion

Extracellular recordings of cells or organs have considerably enriched our knowledge about their function as single units or in networks. They have provided important operational medical devices and have considerable further potential for future applications.^[^ ^2^ ^]^ Within this field, organic bioelectronics are especially promising in view of their potential chemical variabilities, tuneable physicochemical characteristics, mixed conducting properties, and plasticity in form factors.^[^ ^10^ ^]^ Variations in OECT geometry, such as vertical OECTs, provide significant improvements in general transistor characteristics useful for biological signal acquisition.^[^ ^12^ ^]^ Our study demonstrates now for the first time the use of these vertical PEDOT:PSS‐based OECTs (vOECT) as biosensors to perform recordings of cells and micro‐organs. In our goal to provide physiologically meaningful quantitative recordings our main findings include: i) electronic means and characterization methodology to overcome unavoidable imperfections in the device production process; ii) carefully chosen polarization protocols under biological conditions; iii) the recording and extraction of uni‐ and multicellular events in a micro‐organ, the endocrine pancreatic islets, with far smaller signal amplitudes than those recorded previously.

vOECTs are known for their high transconductance exceeding those of planar devices by about a factor of 5, while simultaneously reducing the spatial footprint.^[^ ^12^ ^]^ The sub‐micrometric size of the channel may increase the risk of electric breakdown, however, and voltage biases had to be carefully adapted. Note that often reported physical characterization parameters are obtained during short (seconds) biasing. In contrast, physiologically meaningful recordings may span continuously from minutes to hours, such as during nutrient stimulation of pancreatic islets in‐vitro, mimicking the effects of a meal and the postprandial period.^[^ ^5^ ^]^ To obtain reliable vOECT function we had to carefully titrate the conditions in different settings and to use electrical parameters clearly below the optimal biasing regime for maximum transconductance. Moreover, a culture of cells on vOECT arrays reduced their performance by introducing an additional resistive layer. This effect persisted even after cell removal, probably due to the shedding of extracellular matrices. However, even under those considerations, the maximum transconductance was still superior to those reported for planar OECTs in cellular applications.^[^ ^14^, ^15^, ^16^, ^17^, ^18^, ^19^, ^20^ ^]^ Note also that most reports on transconductances for planar OECTs used in cellular studies have not addressed this issue and it is often not always clear whether reported values correspond to characterizations in the absence or presence of cells, or the extent and duration of previous polarizations. Our exploration of these issues stresses the importance of carefully controlling those parameters in order to obtain quantitatively reliable data over the entire recording period. In the same respect, homogeneity of electrical bias is equally important for biological recordings as small differences in performance between OECT channels may result in different amplification of the signal of interest; this leads to errors in the determination of frequencies due to missing events and errors in the comparison of amplitudes. Detailed and quantitative electrophysiological work on cell signalling and activity using OECTs is still missing despite the multiple demonstrations of their potential usefulness. According to previous studies the maximal transconductance *g_max_* may vary within an OECT chip by a factor of 1.2 to 5 and often this source of error in biological recordings has not been reported.^[^ ^17^, ^18^, ^22^ ^]^ Clearly the development of tuneable boards here has resolved a bottleneck.

The vOECT geometry makes them especially suitable for the future generation of high‐density electrophysiological probes where each probe matches a single cell to obtain crucial spatial information.^[^ ^38^, ^39^ ^]^ Note that so‐called high‐density MEAs consist essentially of a multiplication of electrodes and thus recordable surface but not a substantial improvement of spatial resolution. Obviously, such a setup will only be meaningful if homogeneity of maximal transconductances will either be ensured during production, which most likely presents a major challenge, or if they will be correctly tuned prior to experiments as in our case.

Interfacing biological substrate and organic materials constitutes another important issue in obtaining reliable data. The physicochemical properties of organic polymer transistors are highly favorable for interaction with living cells and organs in terms of tissue reactions and damage.^[^ ^13^, ^40^ ^]^ Indeed, PEDOT:PSS has also been proven innocuous in tests on insulin‐secreting cells.^[^ ^41^ ^]^ In addition, transient influences of the electrically biased polymer on cell activity have to be ruled out. Interestingly, when using different electric biases we did not observe any change in frequency or signal propagation of the biological signals in excitable cells such as cardiomyocytes or islet cells. In contrast, recorded signal amplitudes varied as expected according to the measured transconductance values. Our data are in line with observations made using direct electrical stimulation as well as concomitant recording via OECTs and optical detection of another cell depolarization‐induced intracellular signal, that is, increase in free cytosolic calcium signals.^[^ ^42^ ^]^ Thus, at least under the conditions we used, it is highly unlikely that electrical activities of the recorded cells were changed by the operational device.

The signal quality obtained from islet cells by recording with vOECTs compare rather favorably to those obtained by another extracellular approach, that is, multielectrode‐arrays (MEA) containing PEDOT:PSS covered electrodes. The unicellular action potentials recorded by vOECTs under glucose stimulation show similar frequency to those published, that is, in the range of 0.5 to ≈4 Hz, whereas the amplitudes captured by vOECTs largely exceed the value of 12 µV reported for PEDOT carbon nanotubes coated electrodes in MEAs which excluded their use of their amplitudes as a robust marker in contrast to amplitudes recorded by vOECTs.^[^ ^5^, ^8^ ^]^ Similarly, the frequency of the multicellular slow potentials is in line with previously reported data, where again the amplitude was considerably larger by a factor of two to three as compared to those previously captured by MEAs.^[^ ^5^, ^35^, ^36^, ^43^ ^]^ SPs are of major importance as they are tightly related to insulin secretion, they are deregulated in pathophysiological conditions and their signature is capable of ensuring normoglycemia in a human in‐silico model of an artificial pancreas as a read‐out of glucose levels.^[^ ^5^, ^44^ ^]^ Thus, the amplifying power of the vOECT clearly improves detection, and in combination with the appropriate tunable electronics, is now useable for experiments on excitable cells or micro‐organs of major medical importance such as islets, known for their small signal amplitude.

## 4 Conclusion

Our data on endocrine islet *β*‐cells considerably expand the usefulness of vOECTs in biological and biomedical applications. This study demonstrates the excellent capacity of vOECT to simultaneously capture rapid signals, such as action potentials, as well as slow signals such as multicellular slow potentials. Our work also clearly demonstrates that not only cells or tissues with high amplitude signal, such as neurons and cardiomyocytes, are accessible to OECTs, but also other electrogenic cells with low amplitude signals and well known for their pivotal role in human homeostasis and in a major disease, that is, diabetes.^[^ ^26^, ^27^ ^]^ The qualities of OECTs in general and of vOECTs in particular, open interesting new possibilities. Non‐invasive monitoring is crucial for physiological long‐term experiments to understand micro‐organ function. Their facile deposition, variability in form factors and biocompatibility may also provide more versatility to microfluidic multi‐organ chips.^[^ ^45^, ^46^ ^]^ Moreover, bridging bioelectronics and human tissue has already provided proof of concept for a number of fascinating future biomedical applications in various pathologies.^[^ ^47^, ^48^, ^49^, ^50^ ^]^ OECTs may find an additional role also in a bioinspired artificial pancreas, based on the nutrient‐stimulated electrical activity of islet cells, for bioelectronic organ replacement.^[^ ^43^, ^44^, ^51^, ^52^ ^]^

## 5 Experimental Section

### vOECT Fabrication and Characterization

The fabrication process has been reported previously.^[^ ^12^, ^14^ ^]^ Prior to cell culture devices were treated with plasma (9.82 W/L) for 2 min to favor cell adhesion as described for microelectrode arrays.^[^ ^53^ ^]^ Electrical characterization was carried out in physiological buffered salt solution containing (in millimolar): NaCl 135, KCl 4.8, MgCl_2_ 1.2, CaCl_2_ 1.2‐1.8, HEPES 10 pH 7.4 adjusted with NaOH) with an Ag/AgCl pellet (Multichannel Systems, Tübingen, Germany) gate electrode. A KEITHLEY 2612B dual channel Source Meter was used along with custom LabVIEW software to carry out polarization measurements. The measurement of drain conductance current (*I*_DS_) with changing *V*_GS_ was used in the calculation of the intrinsic transconductance, *g_m_* = Δ*I*_DS_/Δ*V*_GS_ and OECT characteristic curves were plotted using Origin software

### HL‐1 Cell Culture

HL‐1 cells were kindly provided by M. Gramlich (RWTH Aachen, Germany) and cultured according to published protocols in Claycomb medium (51800C, Sigma‐Aldrich, Germany) supplemented with 10% fetal bovine serum (FBS) (v/v) (Eurobio, Courtaboeuf, France), 100 U mL^−1^ penicillin and 0.01% (w/v) streptomycin (Invitrogen, Saint Aubin, France), 0.1 mm norepinephrine (Sigma‐Aldrich, Germany), and 2 mm L‐glutamine (EMD Millipore, Germany).^[^ ^28^, ^54^ ^]^ The chip surface was coated at 37 °C for 1 h with 0.02% w/v gelatine (EMD Millipore, Germany) and 0.1% w/v fibronectin (F‐1141, Sigma‐Aldrich, German). Cells were seeded as 50 000 cells/chip and electrophysiological recordings were performed 6 days later at confluency.

### Islet Isolation and Cell Culture

Islets from adult male C57Bl/6J mice (10–15 weeks of age) were obtained as described.^[^ ^5^, ^35^, ^36^ ^]^ Chip surfaces were coated with Matrigel (2% v/v; BD Biosciences, San Diego, CA, USA) and 100–200 islets were seeded at 37 °C (5% CO_2_, >90% relative humidity) in RPMI medium (11 mm glucose, Thermo Fisher Scientific, Waltham, MA, USA) for 5–6 days on OECTs as entire or as partially dissociated islet‐cell clusters (>10 cells per cluster). All experimental procedures were approved by the Ministry of Education and Research (no. 0 4236.01). To cultivate islets in a small volume, a homemade microfluidic approach was developed using a polydimethylsiloxane (PDMS) microwell, 3 mm in diameter and 3 mm high. The PDMS was cross‐linked at 10% and baked for 1 h at 100 °C before attaching it to the vOECT.

This chip consisted of a polydimethylsiloxane (PDMS) microwell 3 mm in diameter and 3 mm high. The PDMS was cross‐linked at 10% and baked for 1 h at 100 °C before attaching it to the vOECT.

### Extracellular Recording Setup

All measurements were performed at 37 °C with an Ag/AgCl wire as a pseudo‐reference electrode in solution. For HL‐1 cells, the culture medium on the devices was replaced 30 min before recordings by physiological buffered salt solution. For islets, extracellular recordings were performed as described.^[^ ^5^, ^35^, ^36^, ^37^ ^]^ The experimental setup is composed of two multichannel and tunable electronic boards designed to characterize the transistor as well as to measure and monitor the biological signals. The first board (termed ROKKAKU) bridges the non‐standard connector of the sensor device to the second board (termed CHOSEI), which controls polarization and signal conditioning. The polarization circuits in CHOSEI allow via potentiometers for the adjustment of the transistor drain bias to the same value. *I*/*V* converter circuits passively convert the drain current signals into voltage signals using 560 Ω resistors connected to the 12 OECT drains. The output connector gives access to all measured signals from both electrodes and OECTs. Multichannel analogue data were acquired using an INTAN system (INTAN RHD2132 Amplifier Board and controller INTAN RHD2000 USB Interface Board; parallel 24 channel signal sampling at 10 kHz/channel, amplifier bandwidth 0.1 to 3000 Hz). Boards were carefully designed to limit electromagnetic interferences and all recordings were performed inside a grounded Faraday cage. Data were analyzed with MATLAB (MathWorks, Cambridge, UK) and Spike2 software (Cambridge Electronic Design Limited, Cambridge, UK).

### Event Frequency Quantification and Filter Analysis

A 10–100 Hz second‐order Butterworth digital filter was used to extract representative traces of HL‐1 signals and to quantify AP frequencies and SNR. For islets, SPs were extracted using a 0.2−4 Hz band‐pass filter, detected using the peak and threshold module of Spike2 (dead time 200 ms); APs were extracted using a 20−700 Hz band‐pass filter, detected using the threshold module of Spike2 (dead time 10 ms).

Parametric analysis of event detection was conducted for AP and SP, where the cut‐off frequencies varied within the range of interest for the given event (20–700 Hz for APs, 0.2–4 Hz for SPs); the orders varied between 1 and 4, and the detection threshold varied according to signal‐dependent properties (adaptive threshold ranging from −6*σ* to +6*σ* for APs, where *σ* is the signal's standard deviation, and absolute threshold ranging from 0.1% to 100% of the signal's peak‐peak amplitude for SPs). For each filter, the threshold‐dependent average event count was traced. A plateau in the average event count indicates a region of confidence where event detection is robust. The filter was chosen to maximize the width of the confidence region.

An alternative method for the estimation of AP frequency was developed taking advantage of the regularity of APs in HL‐1 cells. For a given electrode, all interspike intervals (ISI) were computed and plotted on a histogram. A Gaussian curve was fitted to the histogram (truncated between 0.6–1.2 s, where the average ISI is expected for HL‐1 cells) using non‐linear least squares fitting solved by the Levenberg–Marquardt algorithm. Although maximum likelihood estimation on a normal distribution would have been best suited in ideal conditions, AP detection in poor SNR conditions results in extraneous ISI peaks near 0 s and at multiples of the average ISI that render maximum likelihood estimation unsuitable. All fits with a coefficient of determination *R* ^2^ < 0.5 were discarded. The average ISI was estimated from the fitted Gaussian curve and inverted to derive the average frequency.

### Signal Propagation Analysis

Signals were down‐sampled to 1 kHz, and AP waveforms were isolated using a narrow 5–20 Hz band‐bass Butterworth filter to minimize noise. APs were then transformed into waveform‐independent spikes using a 100 ms moving RMS filter. Rolling window analysis (10 s window, 75% overlap) of cross‐correlation between all pairs of signals was then performed to extract the time delay between trains of spikes (defined as the time offset where cross‐correlation is maximum, provided correlation at this point was greater than 0.7). The earliest spiking electrode in each window, or “leader”, was identified, and the sequence of the following trains of spikes was determined by sorting the time delays relative to the leader.

### Statistics

Results are presented as means and SEM. Following normality tests, Student's *t*‐test was used for paired data. ANOVA with Tukey as a post hoc test were used for comparisons between more than two groups.

## Conflict of Interest

The authors declare no conflict of interest.

## Author Contributions

M.A. and A.P. contributed equally. Conceptualization: J.L. and S.R.; Data curation: J.L. and S.R.; Formal analysis: M.A., A.P., D.M., G.N., M.R., and J.L.; Funding acquisition: S.R. and J.L.; Investigation: M.A., A.P., M.J.D., D.M., E.P., M.R., G.N., and GP; Project administration: J.L.; Supervision: J.L., S.R., R.O., and M.J.D.; Software: A.P.; Writing – original draft: M.A., D.M., A.P., and J.L.; reviewed by all authors.

## Supporting information

Supporting Information

## Data Availability

The data that support the findings of this study are available from the corresponding author upon reasonable request.
