# Supplementary material for: Vertical Organic Electrochemical Transistors and Electronics for Low Amplitude Micro‐Organ Signals
Source: Adv Sci (Weinh). 2022 Jan 22;9(8):2105211. doi: 10.1002/advs.202105211 (PMC8922095; doi:10.1002/advs.202105211)
Supplement: Supplementary file 1 — Supporting Information [file ADVS-9-2105211-s001.pdf]

ADVANCED SCIENCE
Open Access

Supporting Information

for *Adv. Sci.*, DOI 10.1002/advs.202105211

Vertical Organic Electrochemical Transistors and Electronics for Low Amplitude Micro-Organ Signals

*Myriam Abarkan, Antoine Pirog, Donnie Mafilaza, Gaurav Pathak, Gilles N'Kaoua, Emilie Puginier, Rodney O'Connor, Matthieu Raoux, Mary J. Donahue, Sylvie Renaud and Jochen Lang**

ADVANCED SCIENCE
Open Access

Supporting Information

for *Adv. Sci.,* DOI: 10.1002/advs.202105211

Vertical Organic Electrochemical Transistor Tuning for Low Amplitude Micro-Organ Signals

*Myriam Abarkan, Antoine Pirog, Donnie Mafilaza, Gaurav Pathak, Gilles N'Kaoua, Emilie Puginier, Rod O'Connor, Matthieu Raoux, Mary J. Donahue, Sylvie Renaud, Jochen Lang**

Supporting Information

Vertical Organic Electrochemical Transistor Tuning for Low Amplitude Micro-Organ Signals

*Myriam Abarkan, Antoine Pirog, Donnie Mafilaza, Gaurav Pathak, Gilles N'Kaoua, Emilie Puginier, Rod O'Connor, Matthieu Raoux, Mary J. Donahue, Sylvie Renaud, Jochen Lang**

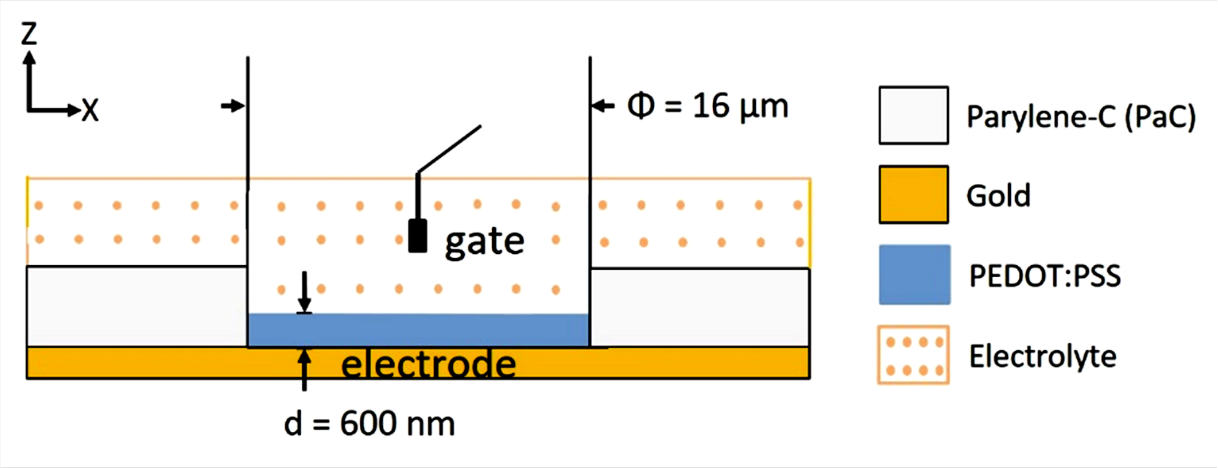

Figure S1. Cross-sectional layout of an electrode and layer dimensions. d, PEDOT:PSS thickness; Φ, electrode dimension. An Ag/AgCl gate electrode is used in characterization and during experiments.

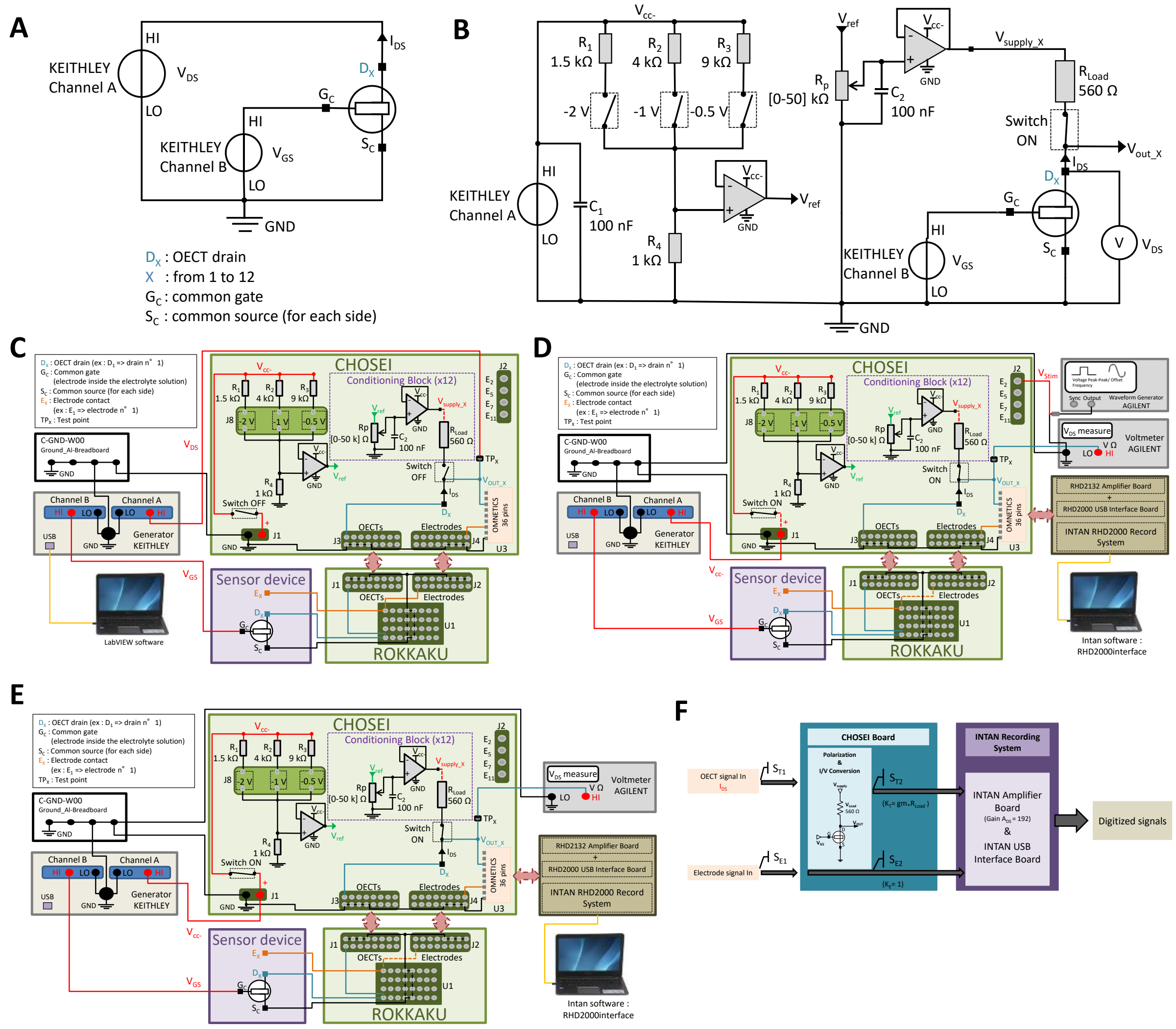

Figure S2. Electronic boards developed for characterization and recording experiments. **A, B.** Scheme of electronic circuits developed for vOECT characterizations and recordings. The drain-source and gate-source voltages are respectively applied to the drain and gate contacts directly. **C.** Setup used for vOECT and electrode characterization experiments. vOECTs and electrodes are connected to the CHOSEI board via the connection board (ROKKAKU). The drain-source bias and gate-source bias from a KEITHLEY source-meter are directly applied to the transistors. Note that biasing circuits and I/V converter circuits were not required for this kind of experiment. Ouput characteristics and transfer curves were measured using a KEITHLEY source-meter and custom LabVIEW program that also plots the data. **D.** Setup for biological signal simulations and validating experiments. An Agilent waveform generator generates standard waveforms (sine, square, pulse) inside the electrolyte bath through one electrode of the device. Input signals are detected by vOECTs before being converted and recorded. **E.** Setup used for electrophysiological experiments with a voltage amplifier and polarization adjustment circuits. The drain-source current is converted into a voltage signal via an I/V

converter circuit while the conditioning circuit adapts the drain-source bias. The source-meter KEITHLEY is used as a DC generator providing gate-source voltage and the power supply voltage for the polarization circuit and the I/V converter blocks. Signals from electrodes and/or vOECTs are conditioned by the voltage amplifier circuit and recorded via an INTAN RHD2000 System (RHD2132 Amplifier Board and RHD2000 USB Interface Board for real-time observation of the signals). The drain-source voltage is continuously monitored by an Agilent voltmeter. **F.** Experimental set up gain and amplification factor. The sensor device contains both vOECTs and PEDOT:PSS coated metal electrodes to compare recordings by the two different technologies.

The signal S_{E1} measured by electrodes is not conditioned by CHOSEI. As a result, the signal collected at the CHOSEI output connector is: $S_{E2} = S_{E1} \times K_E$, with a unitary gain $K_E = 1$. In contrast, voltages S_{T1} sensed by vOECTs generate a current dependent of the vOECT's transconductance *(g_m)*. These signals undergo a I/V conversion resulting in voltages $S_{T2} = S_{T1} \times K_T$ where $K_T = g_m \times R_{Load}$ (where R_{Load} = 560 Ω is the load resistor of the I/V conversion circuit). For a typical transconductance value of 10 mS, $K_T =$ 5.6. All signals are then digitized by the INTAN RHD2132 board (with an on-board gain of 192). They are finally transferred via SPI to the INTAN RHD2000 interface board, where they are made available for recording via USB.

Table S1. Specifications of the CHOSEI board.

<table>
<tr><th colspan="6">Specifications of CHOSEI</th></tr>
<tr><th>Name</th><th>Description</th><th colspan="3">Value</th><th>Unit</th></tr>
<tr><th colspan="6">Voltages specifications</th></tr>
<tr><td rowspan="2">V_{cc-}</td><td rowspan="2">Power supply voltage</td><td colspan="2">Operating voltage</td><td>Max</td><td rowspan="2">V</td></tr>
<tr><td colspan="2">-5</td><td>-15</td></tr>
<tr><td rowspan="2">V_{ref}</td><td rowspan="2">AOP input voltage</td><td colspan="3">Available voltages (V_{cc-} = -5 V)</td><td rowspan="2">V</td></tr>
<tr><td>-0.5</td><td>-1</td><td>-2</td></tr>
<tr><td>V_{supply_X}</td><td>OECT polarization voltage</td><td colspan="3">[0 – V_{ref}]</td><td>V</td></tr>
<tr><th colspan="6">Used components</th></tr>
<tr><td rowspan="5">U4/U5/U6/U8</td><td rowspan="5">Operational Amplifiers (TL08xx) as voltage followers</td><td colspan="2">Operating voltage</td><td>Max</td><td rowspan="5">V</td></tr>
<tr><td colspan="3">V_{cc-}</td></tr>
<tr><td colspan="2">5</td><td>-15</td></tr>
<tr><td colspan="3">V_{cc+}</td></tr>
<tr><td colspan="3">0 (ground)</td></tr>
<tr><td>R_P</td><td>Voltage adjustment potentiometer (conditioning block (x12))</td><td colspan="3">[0-50 k]</td><td>Ω</td></tr>
<tr><td>R_{Load}</td><td>Load resistance (I/V converter circuits)</td><td colspan="3">560</td><td>Ω</td></tr>
<tr><td>TP_X</td><td>Test point for OECT's V_{DS} voltage</td><td colspan="3">12</td><td></td></tr>
<tr><td>J2</td><td>Terminal block (inputs : stimulating electrodes)</td><td colspan="3">4 positions</td><td></td></tr>
<tr><td>J3</td><td>Header test male (inputs : OECTs signals)</td><td colspan="3">2 x 8 positions</td><td></td></tr>
<tr><td>J4</td><td>Header test male (inputs : Electrodes signals)</td><td colspan="3">2 x 8 positions</td><td></td></tr>
<tr><td>U3</td><td>OMNETICS connector (outputs signals: OECTs + Electrodes)</td><td colspan="3">2 x 18 positions</td><td></td></tr>
</table>

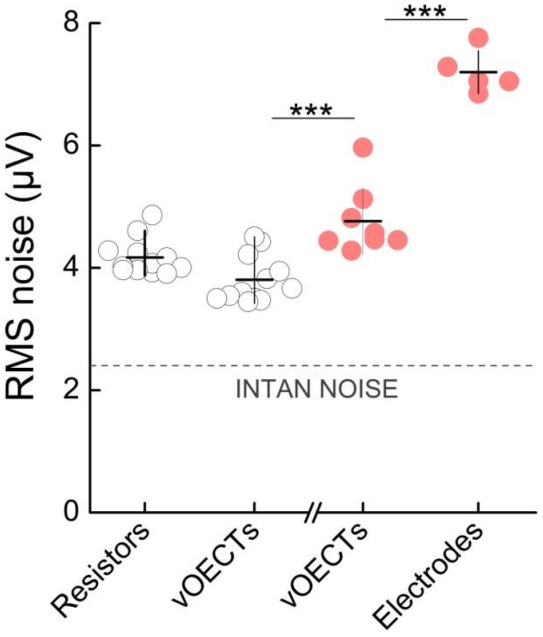

Figure S3. Electronic noise of the set-up. Noise was measured either (left-hand side, open symbols) with resistors of values equivalent to the drain-source junction of the vOECTs and compared with dry vOECTs or with wet setup (right-hand side, closed symbols) of vOECTs or electrodes seeded with HL-1 cells and measured in buffer. The Intan RHD2132 preamplifier's announced input-referred noise is also indicated. The equivalent resistance of every vOECT channel was evaluated by sweeping the drain-source voltage V_{ds} from 0 V to -0.4 V by steps of -0.05 V and measuring the resulting drain current I_{ds}. The equivalent resistance $R_{eq} = V_{ds}/I_{ds}$ was derived through least squares regression for every channel. Resistors of equivalent values were the put together by associating up to three through-hole silicon resistors in series to minimize error. These were then connected between the common source and the drain connector of their corresponding recording channel to model the drain-source junction of the vOECTs. The "drain-source" voltage was adjusted to -0.2 V for each resistor individually as is the case for biological recordings. All 12 channels were recorded simultaneously for 300 s. All resistors were removed and the actual vOECTs were connected in their place using their dedicated connector. The drain-source voltage was adjusted to -0.2 V for each vOECT individually and again, all channels were recorded simultaneously for 300 s.

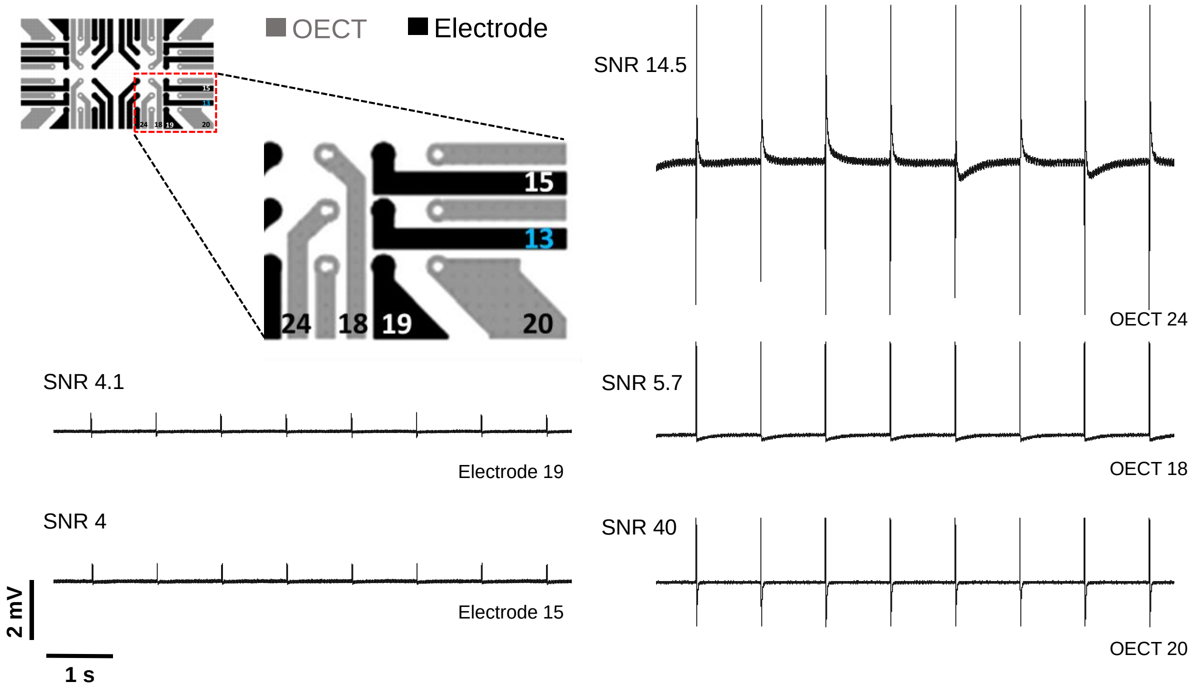

Figure S4. Signal simulation for validation of the setup. The layout of the vOECT/MEA CHIP is shown (upper left) and recorded signals with corresponding numbers of OECT channels or electrodes as well as calculated SNRs. Electrical signals (200 mV, 10 ms, 1 Hz) were applied via electrode 13.

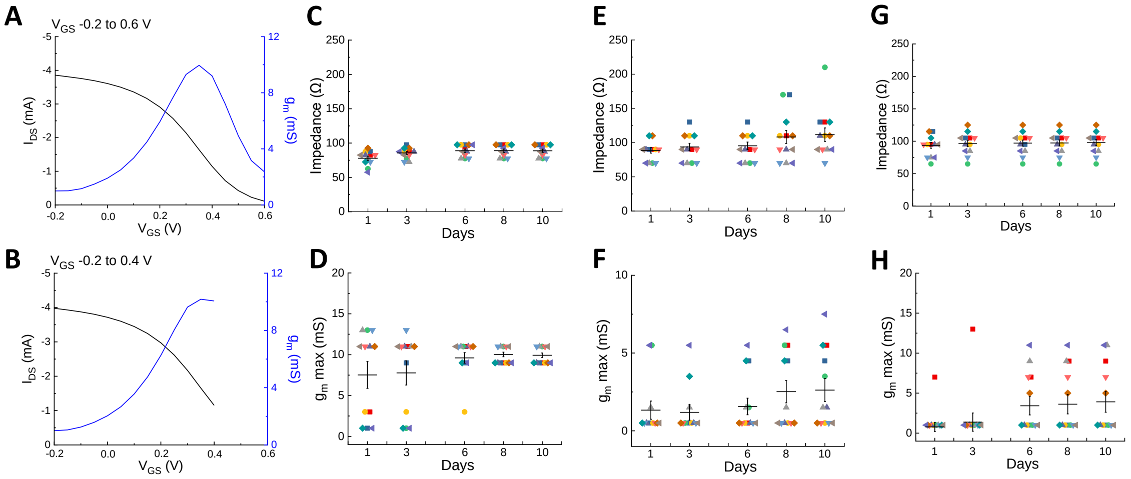

Figure S5. Stability of vOECT performance. Transfer curves and resulting transconductance at V_{DS} = -0.4 V in 0.1 M KCl for V_{GS} **(A)** varying from -0.2 V to 0.6 V or (**B**) varying from -0.2 V to 0.4. Impedance (**C-G**) and $g_{m,max}$ (**D-H**) during repetitive short measurements over days (V_{GS} -0.2 V to 0.4, sufficient to determine $g_{m,max}$, and without polarization in between measurements). vOECTs were kept in physiological buffered salt solution (C,D), in culture medium at 37°C (E,F) or in culture medium after coating of vOECTs with cell adhesion matrix (Matrigel; G,H) as used in recordings of attached cells. Measurements in C-H performed on each vOECT channel are given by different symbols and colors. Statistics did not indicate any significant differences.

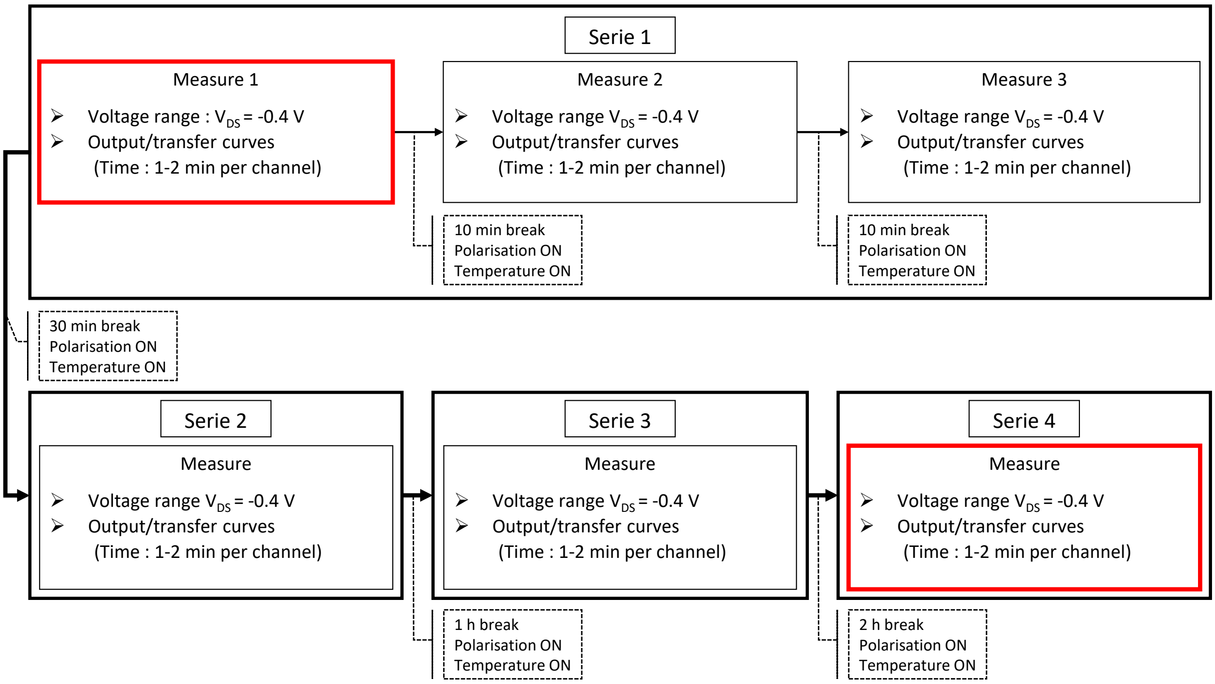

Figure S6. Experimental scheme of measurements given in Fig. 3. 1st and 2nd measurements in Figure 3 correspond to "Measure 1" and "Measure 2"; t0 corresponds to "Serie 1, Measure 1" and t4 h to "Serie 4". For the sake of clarity only these points are depicted in Fig. 3.

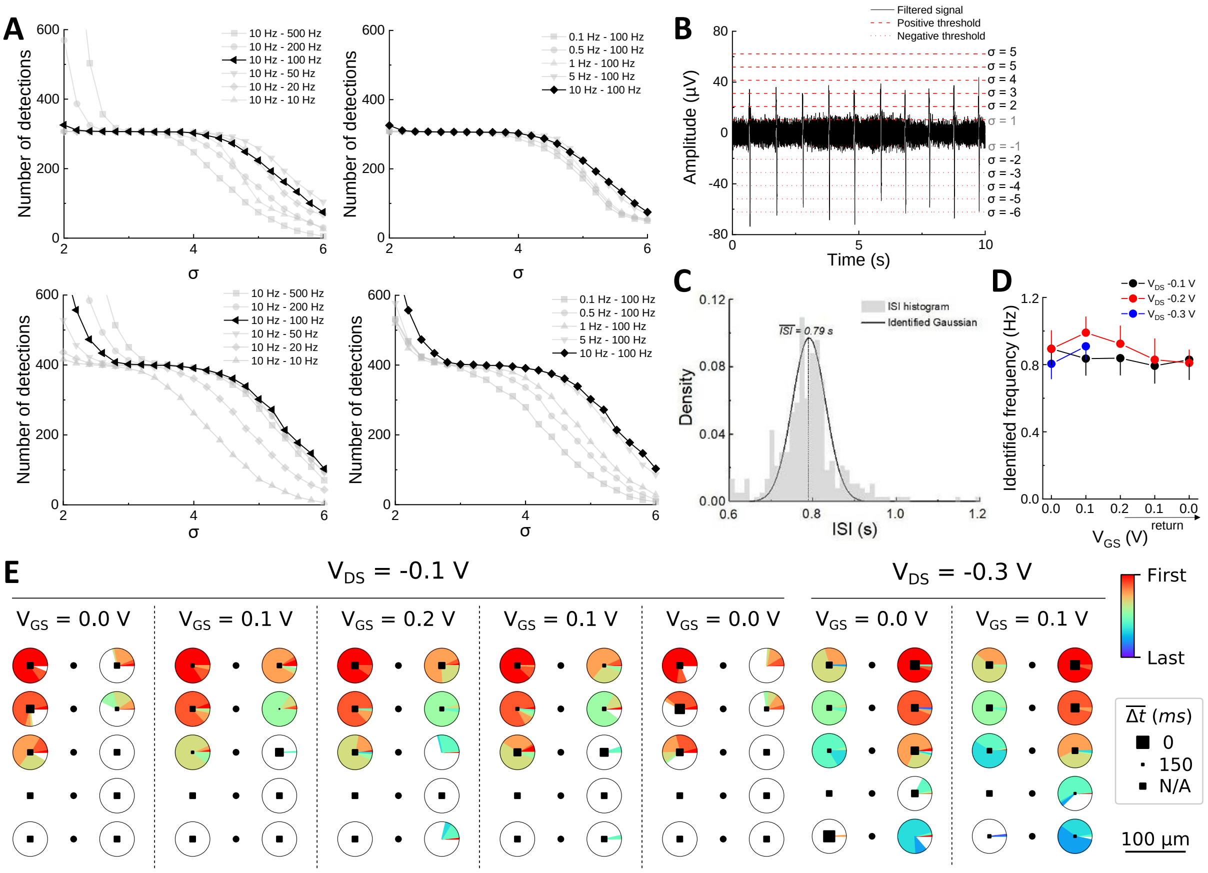

Figure S7. Analysis of signals recorded from HL-1 cardiomyocytes. **A.** Parametric analysis of filters used to detect and extract action potentials recorded by metal electrodes (top) or by vOECTs (bottom). Detections by the combination of a high pass filter of 1 Hz and low pass filter of 100 Hz is given in black, was stable corresponding to a large span of the adaptive threshold σ and was used for all results shown; other combinations are depicted in grey. **B.** Representative trace of several adaptive thresholds σ for action potential detection. σ = -1 or 1 are not adapted because they are too close to the baseline thus picking false positives. **C.** Frequency evaluation method via the interspike interval ISI. The identified Gaussian shows a stable ISI during the recordings. **D.** Frequencies, identified using the ISI method (in D), are stable throughout the electrical conditions. **E.** Action potential propagation across the surface of the chip. Circle symbols represent electrodes, and square symbols represent vOECTs. Solid symbols indicate that the OECT or electrode recorded action potential activity. For each electrode, a pie chart indicates the distribution of spiking order in all measurement windows, and the size of the marker indicates the average delay of spiking relative to the leader.

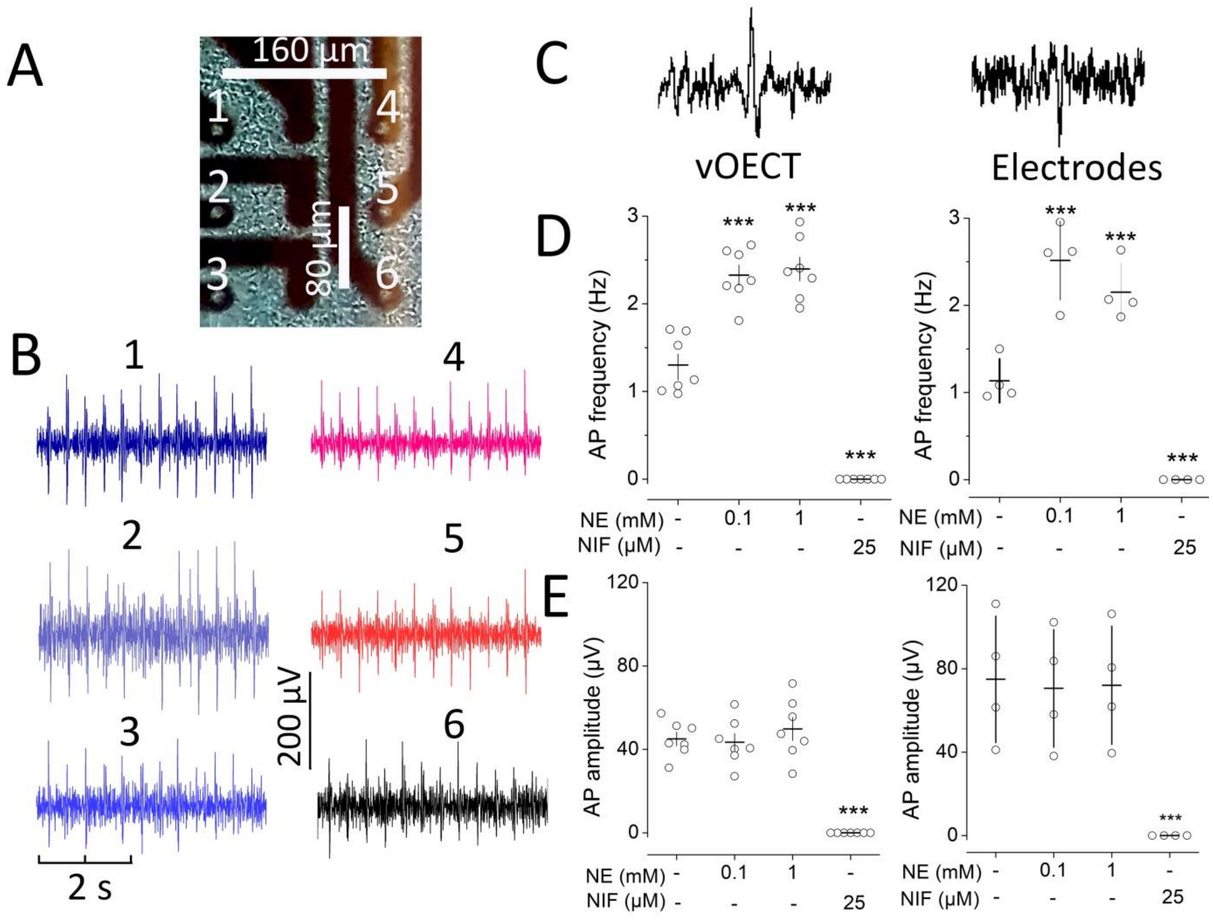

Figure S8. Rhythmicity of clonal HL-1 cardiomyocytes and their regulation by norepinephrine and calcium channel blocker. **A**: Detail and geometry of an OECT with 6 channels. **B**: Recording of HL-1 cells in the presence of 1 mM norepinephrine, channel numbers correspond to those in A. **C**: representative traces of recording via vOECT channels or via electrodes on the same chip. **D** and **E**: Action potential frequency (D) and amplitude (E) of vOECT (left panels) and MEA recordings (right panels). NE, norepinephrine, NIF, calcium channels blocker nifedipine, given are means and SEM; ***, 2p<0.001 (Tukey post-hoc) vs. absence of drugs.

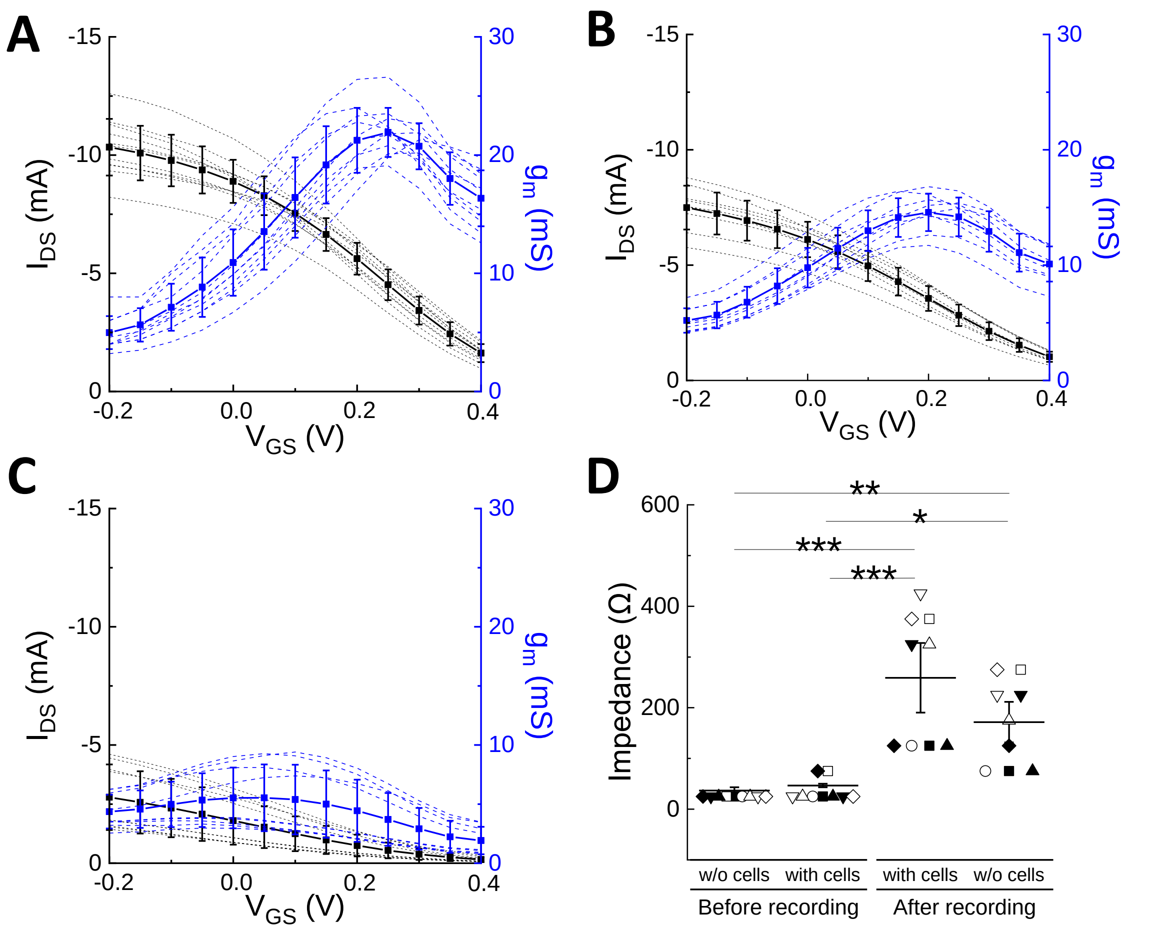

Figure S9. Performance of vOECTs before, during and after islet cell experiments. **A.** Transfer curves and resulting transconductances at V_{DS} -0.4 V, for V_{GS} varying from -0.2 V to 0.4 V in physiological buffered salt solution before seeding islet cells on the vOECTs array, means ± SEM, N = 11, **B.** same as A but with islet cells seeded in culture medium on vOECTs array, means ± SEM, N = 9, **C** same as B but after removal of cells and vOECTs kept in physiological buffered salt solution, means ± SEM, N = 9. **D.** Impedance before recording without cells (buffer, as in A), with cell seeded (culture medium, as in B), directly after recording with cells in culture medium or after removal of cells and addition of physiological buffered salt solution (as in C). Means ± SEM; ANOVA and Tukey's post-hoc analysis; *2p < 0.05, **2p < 0.01, ***2p < 0.001; N = 9-11.

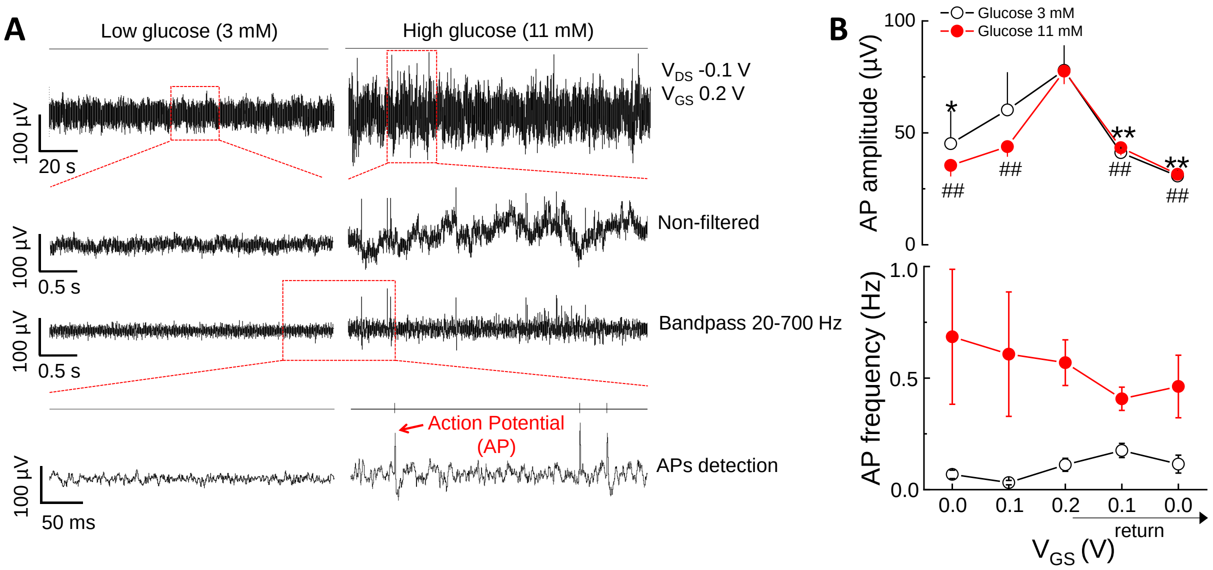

Figure S10. Analysis of signals recorded from pancreatic islets. **A.** Representative raw and filtered recordings of islets at low glucose (non-stimulatory, 3 mM) and high glucose (stimulatory, 11 mM) in physiological buffered ion solution at V_{DS} = -0.1 V and V_{GS} = 0.2 V. Given are different time scales as well as non-filtered and band pass filtered traces (20-700 Hz). **B.** Action potential amplitudes and frequencies at V_{DS} = -0.1 V and indicated V_{GS} sweep. Islets on vOECTs were exposed to low (3mM) glucose or stimulatory concentrations (11 mM). Means ± SEM; paired t test; ## (11 mM) or ** (3 mM) 2p < 0.01 as compared to V_{GS} = -0.2 V, * (3 mM) 2p < 0.05 as compared to V_{GS} = -0.2 V; N = 7. Note that no significant differences in frequencies were observed between all measurements at 3 mM glucose or between all measurements at 11 mM glucose (ANOVA).

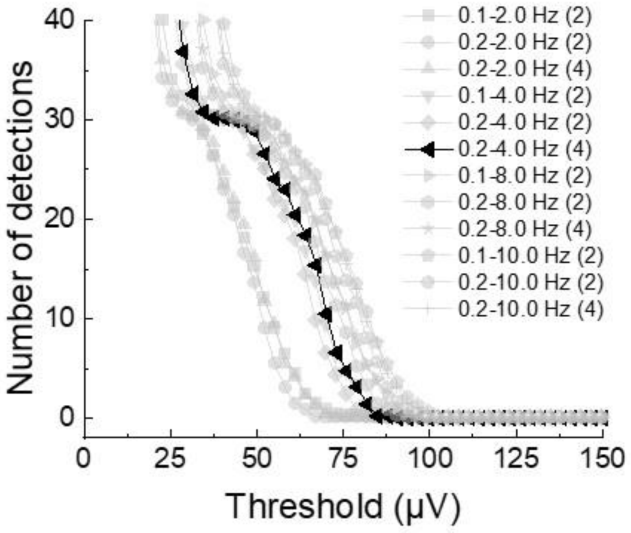

Figure S11. Analysis of signals recorded from pancreatic islets. Parametric analysis of filters used to detect and extract slow potential recorded by transistors. The detection of slow potential is robust at the combination of a 0.2 Hz high pass filter (1st order) and a 4 Hz low pass filter (4th order), given in black, corresponding to a large range of thresholds ranging from 30 µV to 50 µV. This combination was used for analysis; other combinations are given in grey.
